# Supplementary material for: SV40 miR-S1 and Cellular miR-1266 Sequester Each Other from Their Targets, Enhancing Telomerase Activity and Viral Replication
Source: Noncoding RNA. 2022 Jul 28;8(4):57. doi: 10.3390/ncrna8040057 (PMC9413689; doi:10.3390/ncrna8040057)
Supplement: Supplementary file 1 [file ncrna-08-00057-s001.zip › ncrna-1835421-Methods.pdf]

non-coding RNA

MDPI

Supplementary file

SV40 miR-S1 and cellular miR-1266 sequester each other from their targets, enhancing telomerase activity and viral replication

Supplementary file includes:

S1 Supplementary Methods

S1 Supplementary Methods

S1.1. Cell cultures

Dulbecco's Modified Eagle Medium (DMEM) supplemented with 10% FBS, 100 units/mL penicillin G, and 0.1 mg/mL streptomycin sulfate was used to maintain HEK293, GP2-293, A549 (derived from humans), CV-1, and COS-7 (derived from *Cercopithecus aethiops*) cells. BxPC-3 (derived from humans) cells were maintained in RPMI-1640 supplemented with 10% FBS, 100 units/mL penicillin G, and 0.1 mg/mL streptomycin sulfate. HDF were cultured in DMEM supplemented with 10% FBS, 1 ng/mL basic fibroblast growth factor, 5 ng/mL insulin, 100 units/mL penicillin G, and 0.1 mg/mL streptomycin sulfate.

S1.2. Construction of mammalian expression vectors and Luciferase reporter vectors

miR-S1 expression vectors

RNAi-Ready pSIREN-RetroQ (pSRQ, Takara Bio USA, Mountain View, CA, USA) was used as a basal plasmid for construction of miRNA-expression vectors. Using *Bgl*II site, U6 promoter region from pSRQ was replaced into immediate early CMV promoter region from pZac expression vector (provided from Dr. James M. Wilson, University of Pennsylvania) and designed as CMV-cont-PGK-puro. Pre-miR-S1 sequence contains *Xba*I site at the 5′ end and *Eco*RI site at the 3′ end were obtained by PCR using SV40 genomic DNA as a template, and then subcloned into CMV-cont-PGK-puro. Resulted construct was designed as CMV-miR-S1-PGK-puro.

Using CMV-miR-S1-PGK-puro, another kind of miR-S1 expression vector was also constructed. CMV-miR-S1-PGK-puro was digested with *Xba*I and *Not*I, and fragment containing pre-miR-S1 sequence was utilized as insert DNA. Next, pQCXIH retroviral expression vector (Takara Bio USA) was digested with *Xba*I and *Not*I, and insert DNA described above was subcloned. Resulted construct was designed as pQCXIH-miR-S1.

miR-1266 expression vector

First, CMV-cont-PGK-puro was digested with *Bam*HI and *Eco*RI. This linear plasmid was ligated with synthetic DNA contains novel multi-cloning site (5′ *Bam*HI-G-*Not*I-*Nru*I-*Cla*I-TTTTT-*Xba*I-*Eco*RI 3′). Using this modified plasmid, the pre-miR-1266 sequence contains *Bam*HI site at the 5′ end and TTTTT-*Xba*I site-*Eco*RI site at the 3′ end was obtained by PCR using genomic DNA from HEK293, and then subcloned into the modified CMV-cont-PGK-puro. Resulted construct was designed as CMV-miR-1266-PGK-puro.

TERT-HA expression vector

Total RNA was isolated from HeLa cells, and cDNAs containing the complete coding sequence and 3′ UTR sequence of hTERT were synthesized and amplified by PCR. PCR products were subcloned into pQCXIP retroviral expression vector (Takara Bio USA) and resulted construct was designed as the pQCXIP-hTERT-3′ UTR. Using pQCXIP-hTERT-3′ UTR as a template, HA-fused coding sequence were inserted between 3′ end of hTERT coding sequence and 5′ end of 3′ UTR by KOD plus mutagenesis kit (TOYOBO). Resulted construct was designed as pQCXIP-hTERT-HA-3′ UTR. Using pQCXIP-hTERT-HA-3′ UTR as a template, PCR was conducted and hTERT-HA-3′ UTR region was amplified. This PCR product was then subcloned into pZac expression plasmid and resulted construct was designed as the pZac-hTERT-HA-3′ UTR.

Luciferase reporter vectors

Using pRL-CMV (Promega) as a template, sequences of miR-138-5p, miR-152-3p, miR-337-5p, miR-434-5p, miR-532-3p, miR-921, miR-1266-5p, miR-4261, and miR-6771-5p, and complementary sequences of full length miR-S1, mutated miR-S1, miR-1266-3p, and miR-1266-5p were inserted into the downstream of luciferase gene by KOD plus

mutagenesis kit (TOYOBO). Resulted construct was designed as pRL-Luc-miR-138-5p, pRL-Luc-miR-152-3p, pRL-Luc-miR-337-5p, pRL-Luc-miR-434-5p, pRL-Luc-miR-532-3p, pRL-Luc-miR-921, pRL-Luc-miR-1266-5p, pRL-Luc-miR-4261, pRL-Luc-miR-6771-5p, pRL-miR-S1 compl., pRL-miR-S1 compl. mt, pRL-Luc-miR-1266-3pCS, and pRL-Luc-miR1266-5pCS, respectively.

3′ UTR of hTERT containing the seed sequence of miR-1266 was amplified by PCR using genomic DNA from HEK293, and then subcloned into pRL-CMV at *Eco*RV site. Resulted construct was designed as pRL-Luc-hTERT-3′ UTR. Using pRL-Luc-hTERT-3′ UTR, mutated 3′ UTR of hTERT-bearing vector was generated by KOD plus mutagenesis kit (TOYOBO). Resulted construct was designed as pRL-Luc-hTERT-3′ UTR mt.

All of vectors were confirmed successful construction by using direct sequencing with a 3500 genetic analyzer (Applied Biosystems).

S1.3. RT-qPCR

For the detection of pulled-down or immunoprecipitated RNA, RNAs (7.5 μL/reaction) were first subjected to a poly (A)-tailing reaction using poly (A) polymerase (New England Biolabs, Ipswich, MA, USA). These A-tailed RNAs were then reverse transcribed using an anchor RT primer or oligo(dT)$_{12–18}$ primer and a high Capacity cDNA reverse transcription kit (Applied Biosystems, Foster City, CA, USA). Using obtained cDNAs, RT-qPCR for miR-1266-5p and TERT mRNA 3′ UTR (including the target site of miR-1266-5p, i.e., CCCTGAG) was performed according to the manufacturer’s protocol of SYBR Premix Ex Taq II (Takara Bio Japan, Shiga, Japan). PCR amplification and data analysis were conducted using the 7500 Fast Real-Time PCR System (Applied Biosystems). The thermocycling protocol was as follows: initial denaturation at 95 °C for 20 s, 50 cycles at 95°C for 3 s, and amplification at 64°C for 30 s. The primer sequences used in this procedure are listed in Supplementary Table 1. In a pull-down experiment, miR-1266-5p levels were quantified using the values of the amplification signals ($1/2^{Ct}$). In the IP experiment, miR-1266-5p and TERT mRNA 3′ UTR levels were quantified by the ΔΔCt method, whose internal control was the Ct value of an isotype mouse IgG-precipitated sample.

For the detection of cellular mRNA or miRNA, RNAs (500 ng/reaction) were reverse-transcribed using an anchor RT primer, an oligo(dT)$_{12–18}$ primer, or a random primer and the high capacity cDNA reverse transcription kit (Applied Biosystems). Using obtained cDNAs, RT-qPCR for large and small T antigens (LSTag) and TERT mRNA was performed according to the manufacturer’s protocol of SYBR Premix Ex Taq II (Takara Bio, Japan). PCR amplification and data analysis were conducted using the 7500 Fast Real-Time PCR System (Applied Biosystems). The thermocycling protocol was as follows: initial denaturation at 95 °C for 30 s, 40 cycles at 95°C for 3 s, and amplification at 60°C for 30 s. LSTag and TERT levels were quantified by the ΔΔCt method, whose internal control was the Ct value of U6 and β-tubulin, respectively. The primer sequences used in this procedure are also listed in Supplementary Table 1.

For the detection of SV40 levels in cells or in culture media, DNA was isolated using the FavorPrep™ Blood/Cultured cell genomic DNA extraction kit (Favorgen, Ping-Tung, Taiwan). The obtained genomic DNA from cells (100 ng/reaction) or culture media (2 μL/reaction) was subjected to RT-qPCR with the specific primers for LSTag (described above). The thermocycling program was as follows: initial denaturation at 95 °C for 30 s, followed by 50 cycles of 95°C for 5 s, 60°C for 30 s, and 72°C for 30 s of amplification. Fold changes of replication and viral release at 3 dpi were calculated by dividing the value of $1/2^{Ct}$ at 3 dpi by the value of $1/2^{Ct}$ at 1 dpi.

S1.4. Antibodies used in immunoblotting

Rabbit antibodies against Ago4 (clone EPR23799-22, Abcam) and TERT (St John’s Laboratory, London, UK), mouse antibodies against Ago1 (clone 2A7, Wako), Ago2 (clone 4G8, Wako), Ago3 (clone 1C12, Wako), HA (clone 4B2, Wako), large T-antigen (clone

pAb416, Abcam), small T-antigen (clone pAb 280, Novus Biologicals, Centennial, CO, USA), and β-tubulin (clone TUB2.1, Sigma, St. Louis, MO, USA) were used as the primary antibodies. Goat anti-rabbit IgG-HRP and goat anti-mouse IgG-HRP (Sigma) were employed as the secondary antibodies. The dilution rates were as follows: anti-Ago1, 1:1,000; anti-Ago2, 1:1,000; anti-Ago3, 1:1,000; anti-Ago4, 1:1,000; anti-HA, 1:1,000; anti-TERT, 1:1,000; anti-large T-antigen, 1:2,000; anti-small T-antigen, 1:2,000, anti-β-tubulin, 1:5,000; anti-rabbit IgG-HRP, 1:5,000; and anti-mouse IgG-HRP, 1:5,000.
