# Supplementary material for: SV40 miR-S1 and Cellular miR-1266 Sequester Each Other from Their Targets, Enhancing Telomerase Activity and Viral Replication
Source: Noncoding RNA. 2022 Jul 28;8(4):57. doi: 10.3390/ncrna8040057 (PMC9413689; doi:10.3390/ncrna8040057)
Supplement: Supplementary file 1 [file ncrna-08-00057-s001.zip › ncrna-1835421-table S1.pdf]

Table S1. Primers for Reverse transcription, RT-qPCR, and RT-PCR used in this study

Name	Sequence (5' to 3')
Reverse transcription for miR RT-qPCR	
Anchor RT primer 1 *	CGACTCGATCCAGTCTCAGGGTCCGAGGTATTCGATCGAGTCGCACTTTTTTTTTTTTTA
miRNAs, TERT, and TERT mRNA 3'UTR RT-qPCR	
miR-1266-5p-F	GGTGCCCTCAGGGCTGTAGAACA
miR-S1-3p-F	GGTGCGCCTGTTTCATGCCC
U6-F	CGCTTCGGCAGCACATATAC
Universal-R	CCAGTCTCAGGGTCCGAGGTATTC
TERT-F	CGGTGTGCACCAACATCTAC
TERT-R	TCTTGGCTTTCAGGATGGAG
TERT 3'UTR-F	AAGGACCCTGGGAGCTCTG
TERT 3'UTR-R	CCATCCAGGTGCAGGGTCC
Reverse transcription for T antigens	
Anchor RT primer 2	CTGATCTAGAGGTACCGGATCCTTTTTTTTTTTTTTT

	Large T antigen (LTag) and small T antigen (STag) RT-PCR										
	LTag-F			AGTGCCTGGGGGAATATTCCTC							
	STag-F			CGTGTGGGTTGATTGCTACTGC							
	Universal-R2			CTGATCTAGAGGTACCGGATCC							
	tubulin-F			CGCAGAAGAGGAGGAGGATTT							
	tubulin-R			GAGAGGAAAGGGGCAGTTGAG							
	*** Anchor RT primer 1 was used for miRNAs qPCR. For TERT mRNA 3'UTR qPCR, oligo(dT) primer was used.**										
